# The impact of femtosecond laser small incision lenticule extraction on accommodative function

**DOI:** 10.4314/ahs.v25i2.42

**Published:** 2025-06

**Authors:** Liwen Li, Lu Liu, Wenjun Gou

**Affiliations:** 1 Department of Ophthalmology, Suining Central Hospital, Suining, Sichuan, 629000, China; 2 Department of Cerebrovascular Diseases, Suining Central Hospital, Suining, Sichuan, 629000, China

**Keywords:** SMILE surgery, Accommodative amplitude, Accommodative flexibility, Positive and negative relative accommodation, Myopia

## Abstract

This retrospective case study assessed the influence of femtosecond laser small incision lenticule extraction (SMILE) on accommodative function. The study included 42 patients from August 2020 to July 2021 at Suining Central Hospital. They were categorized into near-vision fatigue (20 cases) and asymptomatic groups (22 cases) post-surgery. The near-vision fatigue group received visual training. Comparing pre-surgery, one week, and one month after surgery, accommodative changes were analyzed using variance and t-tests. The near-vision fatigue group had lower preoperative accommodative function (PAPM=0.16, PAF<0.001, PPRA=0.009). One-week post-surgery, they experienced further declines (PAPM=0.021, PAF=0.038, PPRA=0.001), but after one month of training, improvements were evident (PAPM<0.001, PBAF=0.005, PPRA<0.001). The asymptomatic group also had temporary declines one week after surgery, recovering by one month. Negative relative accommodation and accommodative response were minimally affected. SMILE surgery briefly reduced accommodative parameters, predominantly in the near-vision fatigue group. Appropriate training effectively alleviated postoperative near-vision discomfort. In conclusion, SMILE surgery affects accommodative function, leading to temporary declines in specific parameters. Adequate training mitigates these effects, enhancing postoperative comfort.

## Introduction

Myopia, as the second leading cause of blindness worldwide, causes serious disruptions to both individuals and society due to its increasing prevalence^1^-^3^. With the development of technology, improvements in living standards, and increasing health demands, more and more myopic patients are inclined to correct their myopia through refractive surgery^4^. Small-incision lenticule extraction (SMILE) is currently the latest representative of corneal refractive surgery. As the research on SMILE continues to deepen and related surgical techniques continue to improve^5^-^7^, patient satisfaction after surgery is increasing^8^. However, some patients still complain of symptoms such as near-vision fatigue or eye discomfort after surgery. Previous studies have reported that corneal refractive surgery has some impact on patients' accommodative amplitude, leading to a temporary decline in accommodative amplitude shortly after surgery, followed by a gradual recovery^9^, but a comparison of other accommodative parameters is lacking. Moreover, changes in accommodative function directly affect patients' visual experiences. Therefore, this study aims to clarify the impact of SMILE surgery on accommodative function and the role of accommodative function training in improving visual experiences for some patients after surgery by comparing the changes in accommodative function before and after surgery for myopic patients. This will provide a reference for better understanding the impact of SMILE surgery on ocular accommodative function and for further improving patients' postoperative visual experiences.

## Patients and methods

### Study Subjects

A retrospective case analysis was conducted. The medical records of patients who underwent SMILE surgery at Suining Central Hospital between August 2020 and July 2021 were collected.

Inclusion Criteria: A All patients were within the femtosecond surgical indication range,following the expert consensus on the standard of femtosecond laser small incision lenticule extraction surgery in China (2018)^10^; Age≥18 years; Diopter range: spherical lens-3.00 -9.00D, cylindrical lens 0∼-3.00D; (3) The diopter fluctuated within ±0.50D in the past 2a years; Discontinuing contact lens wear, soft contact lens discontinuing for >1 week, hard contact lens discontinuing for >1 month; Informed consent was obtained from patients and their families.

Exclusion criteria: patients with organic diseases; Contraindications to surgery; Patients with other eye diseases; People with immune system diseases; Patients with active ocular inflammatory lesions; Patients with anterior and posterior eye lesions affecting visual function.

Patients who met the surgical indications underwent a comprehensive binocular accommodative function examination before surgery, including accommodative response, accommodative amplitude, positive and negative relative accommodation, and accommodative sensitivity. For patients witaccommodative dysfunction affecting normal vision and binocular visual function, it was recommended to undergo accommodative training first, followed by surgery after symptom resolution. Patients who had complete preoperative and postoperative accommodative function examinations were included in the study to analyze the impact of SMILE surgery on accommodative function. Based on the self-reported symptoms at the one-week postoperative follow-up, patients were further divided into an asymptomatic group and a visual fatigue group. The cause of visual fatigue symptoms and the impact of accommodative training on visual experience and accommodative function were analyzed in conjunction with the patients' preoperative accommodative function. The study was approved by the ethics committee of our hospital

## Methods

### Ocular Examination

All patients underwent a refractive status and accommodative function examination by experienced optometrists before SMILE surgery. The refractive status was determined by adding 0.25D to the power at which the patient's vision showed no significant change with ±0.25D increments. The best-corrected visual acuity was determined based on the patient's ability to recognize a whole row of projected single-line visual targets. Accommodative function examination: All examinations were performed under refractive correction and good lighting conditions, with accommodative amplitude, accommodative response, and positive and negative relative accommodation measured on a comprehensive optometry instrument, and a near visual acuity chart fixed at 40 cm in front of the instrument. The negative lens method was used to measure accommodative amplitude. The BCC method was used to measure the accommodative response. A ±2.00D flipper lens combined with a near visual acuity chart was used to measure the patient's accommodative amplitude. Data from the left and right eyes were measured separately and used for statistical analysis.

### SMILE Surgery

Patients wearing soft or rigid corneal contact lenses were instructed to discontinue use for 2 weeks or 4 weeks, respectively. Three days before surgery, they began using levofloxacin eye drops (National Drug Approval No. H20103148, 5 mL: 24.4 mg) 4 times/day. Routine disinfection and draping were performed, and surface anesthesia was achieved with proparacaine hydrochloride eye drops (National Drug Approval No. H20103352, 0.5%). The VisuMax femtosecond laser surgery system was used for the procedure. The patient was instructed to lie supine and fixate on a fixation light. After the corneal center was aligned, negative pressure suction was applied to stabilize the eyeball. A corneal cap was created using laser pulses with a frequency of 500 Hz and an energy of 120-140 nJ, with a thickness of 120 µm and a diameter of 7.50 mm. Then, a lenticule was created with a base thickness of 15 µm and an optical zone diameter of 6.5mm. A side cut was made at a 90° angle. Scanning was performed in the order of the posterior surface, side cut, and anterior surface of the lenticule. A 3 mm micro-incision was made at the 11:00 position, and a microseparator was used to separate the lenticule from the corneal tissue and remove it. The corneal stromal bed was irrigated with balanced salt solution to complete the surgery.

### Statistical Analysis

The collected data were analyzed using Statistic Package for Social Science (SPSS) 18.0 for statistical analysis (IBM, Armonk, NY, USA). Data that followed a normal distribution were expressed as mean ± standard deviation (), and comparisons were made using the t-test or analysis of variance. Data that did not follow a normal distribution were expressed as median ± interquartile range (m±q), and non-parametric tests were used for comparison. Two-tailed tests were used, and a P-value of less than 0.05 was considered statistically significant.

## Results

### General Situation Comparison

The changes in patients' refractive status and visual acuity before SMILE surgery and one week after the surgery are shown in Table 1. After SMILE surgery, both the spherical and cylindrical refractive errors of the patients were well corrected. The difference between preoperative and one-week postoperative refractive status was statistically significant (P<0.001). There was no significant difference in uncorrected visual acuity one week after surgery compared to preoperative corrected visual acuity, indicating that the effect of SMILE surgery in correcting myopia is excellent and all patients can achieve satisfactory results without glasses after the surgery.

**Table 1:** Refractive status and visual acuity changes of patients before SMILE and at one week after the procedure

Inspection items	Before surgery	One week after surgery	*t*	*P*
Spherical mirror (D)	4.149±1.503	0.014±0.489	23.457	< 0.001
Column mirror (D)	0.863±0.694	-0.234±0.435	11.502	< 0.001
Axial (°)	87.5±170	60.5±146	-1.661	0.097
Visual acuity ((log(MAR))	0.012±0.022	0.011±0.025	18.773	< 0.001

### Refractive Status Comparison

According to the patients' self-reported symptoms at the one-week postoperative follow-up, they were divided into an asymptomatic group and a near-vision fatigue group. We found that there was no significant difference in preoperative refractive status between the two groups (P>0.05). After surgery, the near-vision fatigue group had residual mild myopic refractive errors, but the difference was not statistically significant compared to the asymptomatic group. The difference in the change of refractive errors between the two groups was not statistically significant (P>0.05)(see Table 2). This suggests that the occurrence of self-reported symptoms is not closely related to changes in refractive status.

**Table 2:** Refractive changes in the two groups of patients

Group	Preoperative refractive error	Postoperative refractive error	Difference before and after surgery
Asymptomatic group	-4.551±1.177	0.064±0.411	4.615±1.173
Near-vision fatigue group	-4.612±1.767	-0.083±0.358	4.324±1.731
*t*	0.185	-1.821	-0.895
*P*	0.854	0.072	0.374

### Overall Changes in Accommodative Function

Table 3 shows the changes in accommodative function for all patients one week before and after SMILE surgery. The preoperative accommodative parameters of the patients were basically normal. One week after SMILE surgery, the accommodative amplitude of the patients was significantly lower than before surgery, and the patients' positive and negative relative accommodation and accommodative flexibility were also affected by the surgery, suggesting that SMILE surgery has an impact on the changes in patients' accommodative function.

**Table 3:** Changes in regulatory function of patients before and one week after surgery

	Adjustment range (D)	Adjustment Flexibility (cpm)	Positive Relative Adjustment (D)	Negative Relative Adjustment (D)	Regulated reaction (D)
Before surgery	6.869±2.176	2.905±3.293	-2.687±1.274	2.254±0.494	0.145±0.433
One week after surgery	5.395±1.664	1.570±2.882	-1.888±0.908	2.133±0.638	-0.165±0.530
*t*	2.513	4.000	-2.760	0.891	0.135
*P*	0.16	0.001	0.009	0.187	0.893

### Changes in Accommodative Function by Symptom Group

Further analysis based on patients' self-reported symptoms found that although the age of the two groups was similar (P>0.05)), the near-vision fatigue group had significantly lower preoperative accommodative amplitude (P<0.05), accommodative flexibility (P<0.05), and positive relative accommodation (P<0.05) compared to the asymptomatic group (see Table 4).

**Table 4:** Comparison of the preoperative regulatory functions of the two groups of patients

Group	Sex(Male/Female)	Age	Adjustment range (D)	Adjustment Flexibility (cpm)	Positive Relative Adjustment (D)	Negative Relative Adjustment (D)	Regulated reaction (D)
Asymptomatic group	12/10	28.73±6.635	8.091±1.859	4.272±3.493	-2.954±0.730	2.204±0.405	0.045±0.263
Near-vision fatigue group	9/11	31.3±7.183	5.525±1.664	1.400±2.062	-2.350±0.708	2.275±0.388	0.175±0.230
*t*	0.382	1.207	-4.695	-3.203	2.716	0.574	-1.688
*P*	0.537	0.235	0.000	0.003	0.010	0.569	0.990

### Changes in Accommodative Function in the Near-vision Fatigue Group

The changes in accommodative function before and after surgery in the near-vision fatigue group are shown in Table 5. The accommodative amplitude, accommodative flexibility, and positive relative accommodation at different time points before and after surgery were different (P<0.05). One week after SMILE surgery, the accommodative amplitude in the near-vision fatigue group was further reduced compared to preoperative levels (P<0;05), resulting in an accommodative amplitude below the normal accommodative demand or reserve for near vision^11^. At the same time, the accommodative flexibility and positive relative accommodation in the near-vision fatigue group also significantly decreased after SMILE surgery (both P<0.05) (Table 5). The decrease in accommodative amplitude and flexibility may be the main cause of near-vision fatigue in patients.

**Table 5:** Changes in the preoperative and postoperative regulatory function of patients in the near visual fatigue group

Time	Adjustment range (D)	Adjustment Flexibility (cpm)	Positive Relative Adjustment (D)	Negative Relative Adjustment (D)	Regulated reaction (D)
Before surgery	5.525±1.664	1.400±2.062	-2.350±0.708	2.275±0.388	0.175±0.230
One week after surgery	4.455±1.664*	0.470±1.793*	-1.788±0.908*	2.133±0.638	0.165±0.530
One month after surgery	7.225±1.279^#^*	6.300±3.147^#^*	-2.750±0.559^#^*	2.500±0.362^#^	0.055±0.500
*F*	538.936	126.045	359.421	1723.851	0.784
*P*	0.001	0.001	0.001	0.001	0.386

Note:

* indicates a statistically significant difference compared to preoperative

# indicates a statistically significant difference compared to one week postoperative

After using ±2.00D flipper and bead training for one month, patients reported relief of near-vision fatigue symptoms. When analyzing the changes in accommodative function at this time, we found that patients' accommodative amplitude and flexibility were significantly increased compared to both one week after surgery and preoperatively (both P<0.05). Meanwhile, patients' accommodative reserve (positive relative accommodation) and accommodative relaxation (negative relative accommodation) also significantly improved (P<0.05) (Table 5). The results suggest that flipper training is effective in promoting the improvement of accommodative function in young patients and in resolving postoperative near-vision fatigue symptoms.

### Changes in Accommodative Function in the Asymptomatic Group

In addition, we also analyzed the impact of SMILE surgery on the accommodative function of patients in the asymptomatic group, as shown in Table 6. The accommodative amplitude, accommodative flexibility, and positive and negative relative accommodation at different time points before and after surgery in the asymptomatic group were also different (P<0.05). One week after SMILE surgery, patients' accommodative amplitude, accommodative flexibility, and positive relative accommodation were significantly lower than preoperative levels (P<0.05). However, one month after surgery, patients' accommodative amplitude, accommodative flexibility, and relative accommodation function gradually recovered compared to one week after surgery, with statistically significant differences (P<0.05) (Table 6).

**Table 6:** Changes in regulatory fraction in the asymptomatic group of patients before and after surgery

Time	Adjustment range (D)	Adjustment Flexibility (cpm)	Positive Relative Adjustment (D)	Negative Relative Adjustment (D)	Regulated reaction (D)
Before surgery	8.091±1.859	4.272±3.493	-2.954±0.730	2.204±0.405	0.045±0.263
One week after surgery	6.034±1.364*	2.070±2.602*	-1.878±0.908*	2.233±0.638	0.065±0.437
One month after surgery	7.522±1.891^#^*	5.181±2.822^#^*	-2.841±0.349^#^	2.34±0.397	0.051±0.450
*F*	818.673	52.513	1101.683	980.062	3.384
*P*	0.001	0.001	0.001	0.001	0.062

Note:

* indicates a statistically significant difference compared to preoperative

# indicates a statistically significant difference compared to one week postoperative

## Discussion

SMILE, as the latest representative in corneal refractive surgery, has been proven to be safe and effective in numerous studies^12^-^14^. In this study, we found that SMILE surgery could almost completely correct patients' myopic refractive errors, including myopia (P<0.001) and astigmatism (P<0.001), and the preoperative corrected visual acuity was well reflected in the postoperative uncorrected visual acuity (P>0.05) (Table 1). Previous studies have reported that SMILE surgery can effectively correct myopia, astigmatism, and hyperopia^13^, and most patients who undergo SMILE surgery show postoperative uncorrected visual acuity similar to preoperative best-corrected visual acuity^15^. Our research is consistent with these studies, further demonstrating that SMILE surgery can be effectively used for the refractive correction of myopia and astigmatism patients.

However, although patients achieved good uncorrected visual acuity and relatively satisfactory visual quality after SMILE surgery^8^, some patients still complained of symptoms such as near vision fatigue or short-lasting near vision, affecting their daily life and work. When we analyzed patients' preoperative and postoperative refractive status, we found that there was no significant difference in the refractive changes and postoperative residual degree for patients with near vision fatigue symptoms compared to those without symptoms (P>0.05) (Table 2), suggesting that the occurrence of near vision symptoms has no significant relationship with the current refractive status changes. Previous studies have confirmed that changes in accommodation are the main causes of visual discomfort^16^, ^17^. When we analyzed patients' preoperative and postoperative accommodative function changes, we found that compared with preoperative values, accommodative amplitude, accommodative flexibility, and relative accommodative function all significantly decreased one week after SMILE surgery (Table 3), indicating that SMILE surgery could cause changes in patients' accommodative function. Our results are consistent with the research of Q-B. Li et al.^9^ and Karimian et al.^18^, which demonstrated that regardless of low or high myopia, significant decreases in accommodative amplitude occurred around 10 days after corneal refractive surgery. Further subgroup analysis revealed that for patients with near vision fatigue symptoms, their accommodative function was significantly lower than that of asymptomatic patients even before surgery (P=0.000) (Table 4). After undergoing SMILE surgery, further decreases in accommodative function (Table 5) led to accommodative amplitude in the near vision fatigue group lower than the required accommodative demand or reserve for near vision^11^, which might be one of the reasons for postoperative near vision fatigue in these patients^19^.

The normal functioning of ocular accommodation is one of the essential factors in maintaining visual comfort. Thiagarajan et al.'s study^20^ showed that accommodation abnormalities are one of the main causes of near vision fatigue, and lens accommodation training can effectively improve visual fatigue symptoms^21^, ^22^. For patients experiencing near vision fatigue symptoms after surgery, we provided appropriate accommodative function training after a reasonable analysis, helping them to recover autonomous accommodation as soon as possible and adapt to the accommodative demands required for near vision. After one month of ±2.00D flipper lens combined with bead string training, the patients' main complaints of near vision fatigue symptoms were alleviated, and further examination of accommodative function showed that their accommodative amplitude and flexibility gradually increased (Table 5), while positive and negative relative accommodation also significantly improved. Liu's study^23^ showed that proper visual training could significantly improve patients' accommodative amplitude, positive relative accommodation, negative relative accommodation, and other accommodative and convergence functions, reducing visual fatigue symptoms and binocular vision symptoms in patients with visual fatigue symptoms and binocular vision abnormalities. Our study is consistent with these findings, suggesting that appropriate visual training can effectively alleviate patients' near vision discomfort symptoms.

Karimian et al.'s study^18^ showed that accommodative amplitude and flexibility decreased in the early stage after corneal refractive surgery but eventually increased gradually. In our study, we found that the accommodative changes in asymptomatic patients were similar to those in Karimian et al.'s study: patients' accommodative amplitude, sensitivity, and positive relative accommodation gradually recovered one month after surgery compared with one week after surgery, suggesting that the impact of corneal refractive surgery on accommodative function is transient (Table 6). Unfortunately, the cause of this change is still unclear, possibly related to the stimulation of corneal anterior surface nerve function^24^.

However, visual discomfort symptoms are not only related to accommodative factors but also closely related to convergence function. This study only observed the short-term effects of SMILE surgery on accommodative function and did not collect data on changes in convergence-related parameters. Nevertheless, our results showed that the decrease in accommodative function after SMILE surgery might be one of the reasons for some patients' postoperative visual discomfort symptoms, and appropriate visual training can effectively help symptomatic patients resolve their discomfort symptoms early after surgery, improving their satisfaction with SMILE surgery.

## Data Availability

The data that support the findings of this study are available from the corresponding author upon reasonable request.
